# What research exists on the presence of 6PPD-Q in different environmental media? A systematic map protocol

**DOI:** 10.1186/s13750-026-00380-1

**Published:** 2026-02-04

**Authors:** Sultan Aljohani, Mary Engels, Kenneth E. Wallen

**Affiliations:** University of Idaho, 875 Perimeter Dr, 83844 Moscow, ID USA

**Keywords:** 6PPD-quinone, Water, Air, Dust, Soil, Sediment, Tire wear particles

## Abstract

**Background:**

Automobiles are ubiquitous in the modern world, and chemicals leaching from car tires and from the tire wear particles produced during driving can be toxic to the environment, particularly in aquatic ecosystems. 6PPD-Quinone (6PPD-Q), a recently identified tire and tire wear particle leachate, has been identified as highly toxic to coho salmon and other aquatic species. Research on the distribution and impacts of 6PPD-Q in aquatic ecosystems is rapidly developing, while research on 6PPD-Q in other environmental media is just beginning. With research efforts developing on many fronts, there is a need to better map emerging knowledge about this toxin. To do that, we ask the question: “What research exists on the presence of the 6PPD-Q in different environmental media (water (freshwater), soil, sediment, and air, including dust)?” The ultimate purpose of this systematic map is to generate a literature catalog that serves as a searchable database about 6PPD-Q in different environmental media.

**Methods:**

The systematic map will follow the Collaboration for Environmental Evidence guidelines and conform to the Reporting Standards for Systematic Evidence Syntheses (ROSES). Relevant English language only literature searches will use a search string using the specified Boolean description of our PECO elements (Population: Environmental media water, soil, sediment, air: including dust; Exposure: N/A; Comparator: N/A; Outcome: The presence of 6PPD-Q/ The concentration of 6PPD-Q). Two bibliographic databases, Web of Science (WOS) Core Collection and ScienceDirect, will be searched. Additional literature will be located through searches of targeted search engines and specialist websites. Screening of titles, abstracts, and full texts will be completed in series using established eligibility criteria. The results of the systematic map will contain a searchable open-access database formatted in Microsoft Excel. Furthermore, the outcome will be presented in a global map of the geographical distribution of included studies and their PICO/PECO elements, including a narrative synthesis, descriptive statistics, tables, and figures.

**Supplementary Information:**

The online version contains supplementary material available at 10.1186/s13750-026-00380-1.

## Background

Automobiles are ubiquitous in the modern world, and chemicals that leach from tires can be toxic to the environment, particularly aquatic ecosystems. Recently, a tire associated chemical found in road runoff, N-(1,3-dimethylbutyl)-N′-phenyl-p-phenylenediamine quinone, or 6PPD-Quinone (6PPD-Q), has been identified as a potential threat to aquatic ecosystems and is known to be highly toxic to coho salmon (*Oncorhynchus kisutch*) and other aquatic species [1–4]. 6PPD-Q forms as a degradation product of N-(1,3-dimethylbutyl)-N′-phenyl-p-phenylenediamine or 6PPD, which is used as an antiozonant and antioxidant in tire production to lengthen and prolong tire lifespan [5]. When the parent chemical, 6PPD, undergoes chemical transformation to water soluble 6PPD-Q it readily leaches into the ecosystem where it can interact with and affect living organisms [5]. Understanding the fate and behavior of 6PPD-Q is therefore crucial, particularly given its water solubility and potential for bioaccumulation in organisms.

The primary way 6PPD-Q gets into the environment is through the creation and distribution of tire wear particles (TWPs). TWPs and tire road wear particles (TRWPs) are tire fragments resulting from the abrasion of tires against the road surface or are mixtures of tire fragments and road materials in the case of TRWPs. These particles, which many studies do not differentiate [4], represent one of the largest sources of microplastics to the environment [6]. Made from materials specifically engineered to resist breakdown, TWPs and TRWPs are long-lived pollutants in the environment and can be found in many different environmental media, including soils, sediment, and air, in addition to water [7]. While 6PPD-Q has been primarily documented in aquatic ecosystems, recent research indicates that 6PPD-Q is also present in many different environmental media [2], though the full distributions are not yet clear. This widespread occurrence underscores the need to systematically characterize 6PPD-Q to assess current environmental and human health risks and to inform evidence-based policy and rulemaking aimed at limiting future contamination.

The rapid growth in 6PPD-Q research necessitates comprehensive evaluation of the current state of knowledge regarding 6PPD-Q presence in the environment and analytical detection methods [8]. Developing and documenting a replicable systematic map protocol would be a valuable addition to the existing body of evidence synthesis literature and support potential future systematic review efforts [9]. To ensure that this development effort was not duplicative, we searched the existing corpus of evidence synthesis literature related to 6PPD-Q in environmental media. We found no relevant articles in databases focused on systematic evidence syntheses (Cochrane Database, Prospero, Epistemonikos, and OSF), though we identified 15 articles classified as reviews in the Web of Science Core Collection (WOSCC) (Additional File 5). Of these articles, seven were ad hoc reviews with no documented review methodology and eight were systematic reviews with varying levels of methodological documentation (Additional File 6).

The primary limitation of the existing systematic reviews is that none of the articles included environmental media specific terms in their search strings or keywords, despite many explicitly discussing environmental distribution, occurrence, or sources within their scope. This inconsistent definition of environmental media limits the utility of existing reviews for researchers focusing on specific environmental compartments. For this reason, we feel it is important to develop a protocol to identify the current state of knowledge related to 6PPD-Q in specific environmental media (water (freshwater), soil, sediment, air) and techniques related to its identification and analysis.

Our aim in developing this protocol is to systematically map and catalog the current peer-reviewed literature on 6PPD-Q in environmental media. This catalog will serve two important purposes: identifying well-evidenced areas suitable for evidence synthesis and meta-analyses, and providing a replicable framework that enables the scientific community to examine, use, and periodically update this resource as new research emerges.

### Definitions

For this protocol, we use the definition of environmental media provided by the Agency for Toxic Substances and Disease Registry: “soil, water, air, biota (plants and animals), or any other parts of the environment that can contain contaminants” [10]. Further, the Natural Resources Conservation Service defines the soils as: “The unconsolidated mineral or organic material on the immediate surface of the Earth that serves as a natural medium for the growth of land plants” [11]. In addition, freshwater is defined as “water containing less than 1,000 milligrams per liter of dissolved solids, most often salt”, which consists of various forms such as rivers, lakes, reservoirs, creeks, streams, and groundwater [12]. Finally, sediments are the “materials found at the bottom of a water body” [13].

### Stakeholder engagement

Due to resource constraints, stakeholder engagement was limited to consultation with a single expert advisor (Dr. Mohamed H. EL-Saeid at King Saud University, KSA). The research question development went through an iterative process. The initial question was developed by researcher SA, who then discussed and revised the question based on feedback from the expert advisor. Further revisions and refinements came from discussion between SA and ME, and the question was finalized with the input of KW, who has significant experience with systematic mapping efforts. The development of the actual systematic map will follow the same protocol for the same resource constraint reasons. This structure is compliant with and recommended by Collaboration for Environmental Evidence (CEE) 5.1 [14].

## Objective of the review

The systematic map aims to measure and profile peer-reviewed and select gray literature sources, investigating the existence of 6PPD-Q in different ecosystems, and which methods (including instruments) are used to collect and quantify 6PPD-Q. While there are some evidence synthesis studies on 6PPD-Q, such as Bohara et al. (2024), based on our knowledge, no systematic maps target 6PPD-Q. Thus, this will be the first systematic map of research on 6PPD-Q, and this mapping will contribute to minimizing the knowledge gaps about where this novel toxin is found in the environment.

### Primary question components

For the question elements of this systematic map/protocol, we followed guidelines established by the CEE [14]. Since the purpose of this systemic map is to identify research on the presence of 6PPD-Q in environmental media, regardless of source, the systemic map question is defined in a PO framework, with only the populations and outcomes specified. Thus, we used the PECO structure elements to segment our primary research question as follows:

P: Population = Environmental media **[**water(freshwater), soil, sediment, air: including dust].

O: Outcome(s) = The presence of 6PPD-Q/ The concentration of 6PPD-Q.

### Primary question

What research exists on the presence of the 6PPD-Q in different environmental media [water(freshwater), soil, sediment, and air: including dust]?

### Secondary questions

1. What is the geographic distribution of 6PPD-Q data in existing literature?
2. What is the geographic distribution of environmental media in the existing literature about 6PPD-Q?
3. Which environmental media are studied the most?
4. Which methods have been used to detect 6PPD-Q in each environmental medium?
5. Which instruments have been used to detect 6PPD-Q in each environmental medium?
6. What is the Extraction Recovery, Limit of Detection (LOD), and Limit of Quantitation (LOQ) for 6PPD-Q (if applicable) within different environmental media?
7. How has the volume of knowledge about 6PPD-Q in different environmental media changed with time?
8. Where do the gaps and aggregates of 6PPD-Q in different environmental media knowledge exist?

## Methods

The systematic map will follow the CEE guidelines and conform to RepOrting standards for Systematic Evidence Syntheses (ROSES) [15]. Under the requirements of the CEE guidelines, this protocol has been registered in the PROCEED database (PROCEED-25-00365, not finalized yet).

### Searching for articles

#### Search terms and language

Searches will be performed using search terms exclusively in the English language. Nonetheless, the result may yield articles written in languages other than English, and studies written in English and Arabic will be included, as one of the authors is a native Arabic speaker. The list of search terms is presented in the next section (See Search string).

#### Search string

Search terms were developed based on the PECO/PO elements described above. Based on scoping exercises (Additional File 2), the following search string was developed (WOSCC) format): TS= ((“6PPD-Q” OR “6PPD-quinone” OR “6PPDQ”) AND (“tire wear particles” OR “tire road wear particles"OR"soil*” OR “sediment*” OR freshwater* OR “fresh water” OR lake* OR ponds* OR river* OR reservoir* OR stream* OR groundwater OR “surface water” OR runoff OR aquat* OR “particulate matter” OR “air” OR “dust” OR “aerosol*” OR “atmospher*”)). Search strings formatted for each source are documented in Additional File 4.

#### Estimating the comprehensiveness of the search

To assess the comprehensiveness of our search and minimize the error, we developed a 24-article Benchmark list considered relevant to the research questions, covering a broad range of the PECO elements (Additional File 1). In addition, scoping tests were conducted on the Web of Science Core Collection and ScienceDirect to examine the search string and inspect the Benchmark list (Additional File 2). All final search strings were documented (Additional File 4).

#### Bibliographic databases

Our principal database for peer-reviewed literature will be the Web of Science Core Collection (WOSCC), accessed via an institutional subscription (University of Idaho). The WOSCC has been characterized as the original global citation index for scientific and scholarly research [16]. Searches will not be restricted by publication year. The second database is ScienceDirect, which we can also access through our institution. According to Gusenbauer and Haddaway (2020), the two databases are adequately suited for applying the evidence synthesis process [16].

#### Internet searches and grey literature

Grey literature will include dissertations and theses, methods papers, and journal articles (non-peer-reviewed). Searches for these documents will focus on various topic specific search engines and websites. We will search ProQuest Dissertations & Theses Global, for which we have Institutional access, for any relevant dissertations or theses. All other literature from ProQuest searches will be excluded. Internet searches for additional grey literature will be conducted using the following websites:

BASE (https://www.base-search.net).

GreenFILE (http://www.greeninfoonline.com).

National Service Center for Environmental Publications (https://www.epa.gov/nscep).

All techniques for all search terms and/or strings, as well as filtering/limitations for grey literature searches, can be found in Additional File 4.

#### Assembling and managing search results

Search results will be collated and duplicates removed. The map will be managed with Rayyan, a specialized tool for systematic evidence synthesis, and the reference management software Zotero (and Zotero Connector).

### Article screening and study eligibility criteria

#### Screening process

Articles will be screened for eligibility in two steps: first on title and abstract, and second on full-text. Articles with unclear eligibility status during title/abstract screening will be included for full text screening, and articles without an abstract or summary (but retained based on title) will be screened on their full-text. Screening will be conducted by at least two members of the review team. Reviewers will use the Rayyan website to complete all screening processes.

To evaluate the consistency of screening decisions between reviewing members, we will use Randolph’s Kappa coefficient on random samples of 10% of the articles for screening at the titles and abstracts stage, and 5% for screening on the full-text stage. We will consider at least a 0.6 coefficient agreement as a satisfactory level between the reviewers. In the case of disagreement, there will be a discussion to resolve the dispute by improving the value of the coefficient [17].

#### Eligibility criteria

The eligibility of articles will be assessed at each stage using the criteria presented in Table 1. A list of articles rejected at full-text screening will be documented, including the reason for exclusion.

**Table 1:** Eligibility criteria

Inclusion criteria	Exclusion criteria
Eligible population	
The systematic map will include only the studies about 6PPD-Q in following parameters: Water (freshwater), such as lakes, ponds, snow, rivers, surface water, groundwater, stormwater, and runoff water. Soil, Sediment, and Air, including dust and aerosols, together or in separate studies.	Any biotic media (e.g. precise organs (liver, kidney, etc.) species, humans, animals, etc.). Saltwater, such as seawater or marine water, will be excluded. Wastewater will be excluded.
Eligible Outcomes.	
Studies that report on 6PPD-Q using any unit of concentration or simple presence or absence.	Studies that do not report on 6PPD-Q specifically.
Eligible Study Designs.	
The systematic map will include experimental, nonexperimental, observational, laboratory, and field studies.	For a comprehensive search, evidence synthesis studies will be excluded due to their limitation criteria.
Eligible Documents.	
Dissertations and Theses, methods papers, journal articles (peer and non-peer-reviewed).	Other, such as: letters or news items, conference Presentations or abstracts, posters, etc.

#### Study validity assessment

The systematic map will not critically appraise the included studies. The mapping effort aims to explore and identify the distribution of 6PPD-Q in different environmental media within the academic literature via a comprehensive narrative synthesis. This will help position future systematic reviews in this field.

#### Data coding strategy

The following list of variables from full-text articles will be documented by the review team (SA) in a Microsoft Excel sheet for inclusion in the map (Additional File 3):

- Bibliographic information.
- Details about the inclusion criteria.
- Details about the study types.
- Details about the locations.
- Details about extraction methods (including the instruments).
- Details about Extraction Recovery, Limit of Detection (LOD), and Limit of Quantitation (LOQ).

Data coding will follow an a priori specified codebook. Before coding, a random selection of 10% of included articles will be coded independently by two members of the review team (SA and ME). Any disagreement will be discussed and resolved, and all reviewers will make necessary adjustments to the coding strategy.

#### Study mapping and presentation

An open-access database formatted in Microsoft Excel of all included studies and coded data will be added as an appendix to the systematic map publication. A narrative synthesis with descriptive statistics, tables, and figures will be presented to describe the characteristics of the reviewed studies. A global map of the geographical distribution of included studies and their PICO/PECO elements will be presented and geographic analysis will be by continent and by region, as articulated in Additional File 3. Within each environmental media, analysis will focus on levels of 6PPD-Q found and methods of detection and their efficacy.

## Supplementary Information

Below is the link to the electronic supplementary material.

Supplementary Material 1.

Supplementary Material 2.

Supplementary Material 3.

Supplementary Material 4.

Supplementary Material 5.

Supplementary Material 6.

Supplementary Material 7.

Supplementary Material 8.

## Data Availability

All data generated or analyzed during this study are included in this published article [and its supplementary information files].

## References

[1] Hiki K, Asahina K, Kato K, et al. Acute toxicity of a tire Rubber-Derived Chemical, 6PPD Quinone, to freshwater fish and crustacean species. Environ Sci Technol Lett. 2021;8:779–84. 10.1021/acs.estlett.1c00453.

[2] Ihenetu SC, Xu Q, Khan ZH, et al. Environmental fate of tire-rubber related pollutants 6PPD and 6PPD-Q: A review. Environ Res. 2024;258:119492. 10.1016/j.envres.2024.119492. 38936499 10.1016/j.envres.2024.119492

[3] Tian Z, Gonzalez M, Rideout CA, et al. 6PPD-Quinone: revised toxicity assessment and quantification with a commercial standard. Environ Sci Technol Lett. 2022;9:140–6. 10.1021/acs.estlett.1c00910.

[4] Zhao T, Zhang Y, Song Q, et al. Tire and road wear particles in the aquatic organisms – A review of source, properties, exposure routes, and biological effects. Aquat Toxicol. 2024;273:107010. 10.1016/j.aquatox.2024.107010. 38917645 10.1016/j.aquatox.2024.107010

[5] Chen X, He T, Yang X, et al. Analysis, environmental occurrence, fate and potential toxicity of tire wear compounds 6PPD and 6PPD-quinone. J Hazard Mater. 2023;452:131245. 10.1016/j.jhazmat.2023.131245. 36958160 10.1016/j.jhazmat.2023.131245

[6] Werbowski LM, Gilbreath AN, Munno K, et al. Urban stormwater runoff: A major pathway for anthropogenic Particles, black rubbery Fragments, and other types of microplastics to urban receiving waters. ACS EST Water. 2021;1:1420–8. 10.1021/acsestwater.1c00017.

[7] Aziz T, Mann A. (2024) The Presence and Potential Impacts of the Tire-Wear-Derived Compound (6PPD-q) on NC Aquatic Ecosystems.

[8] Bohara K, Timilsina A, Adhikari K, et al. A mini review on 6PPD quinone: A new threat to aquaculture and fisheries. Environ Pollut. 2024;340:122828. 10.1016/j.envpol.2023.122828. 37907191 10.1016/j.envpol.2023.122828

[9] Ralph P, Baltes S. (2022) Paving the Way for Mature Secondary Research: The Seven Types of Literature Review. In: Proceedings of the 30th ACM Joint European Software Engineering Conference and Symposium on the Foundations of Software Engineering. pp 1632–1636.

[10] ATSDR. (2025) Glossary | PHA Guidance Manual. https://www.atsdr.cdc.gov/pha-guidance/glossary/index.html. Accessed 9 July 2025.

[11] Natural Resources Conservation Service Soil Facts. https://www.nrcs.usda.gov/resources/education-and-teaching-materials/soil-facts. Accessed 8 July 2025.

[12] U.S. Geological Survey. (2019) Freshwater (Lakes and Rivers) and the Water Cycle. https://www.usgs.gov/special-topics/water-science-school/science/freshwater-lakes-and-rivers-and-water-cycle. Accessed 9 July 2025.

[13] US EPA. (2015) Superfund: Contaminated Sediments. https://www.epa.gov/superfund/superfund-contaminated-sediments. Accessed 8 July 2025.

[14] Collaboration for Environmental Evidence. (2022) Guidelines and Standards for Evidence synthesis in Environmental Management. Version 5.1 (AS Pullin, GK Frampton, B Livoreil&G Petrokofsky, Eds).

[15] Haddaway NR, Macura B, Whaley P, Pullin AS. ROSES reporting standards for systematic evidence syntheses: pro forma, flow-diagram and descriptive summary of the plan and conduct of environmental systematic reviews and systematic maps. Environ Evid. 2018;7:7. 10.1186/s13750-018-0121-7.

[16] Gusenbauer M, Haddaway NR. Which academic search systems are suitable for systematic reviews or meta-analyses? Evaluating retrieval qualities of Google Scholar, PubMed, and 26 other resources. Res Synth Methods. 2020;11:181–217. 10.1002/jrsm.1378. 31614060 10.1002/jrsm.1378 PMC7079055

[17] Ouédraogo D-Y, Sordello R, Brugneaux S, et al. What evidence exists on the impacts of chemicals arising from human activity on tropical reef-building corals? A systematic map protocol. Environ Evid. 2020;9:18. 10.1186/s13750-020-00203-x.

