# Supplementary material for: What research exists on the presence of 6PPD-Q in different environmental media? A systematic map protocol
Source: Environ Evid. 2026 Feb 4;15:2. doi: 10.1186/s13750-026-00380-1 (PMC12922382; doi:10.1186/s13750-026-00380-1)
Supplement: Supplementary file 2 — Supplementary Material 2. [file 13750_2026_380_MOESM2_ESM.docx]

**Additional File 2: Search string development.** Details of the scoping exercise performed to build the search string.

For the purpose of the scoping exercise to build and develop the search string, we performed searches on the Web of Science Core Collection (WOSCC) database using the term (TS). We had access to the following WOS CC Citation Indexes: Science Citation Index Expanded(1900-present); Social Sciences Citation Index (1900-present); Arts & Humanities Citation Index (2005-present); Emerging Sources Citation Index (2005-present).

The search string was constructed through a multi-scoping exercise, documented in the table below. The first scoping exercise was conducted in April 2025, and the search string was further revised in May 2025 and then in July 2025. Following a meeting with all authors to discuss validation of the protocol and the search string, the search string was finalized in the last part of the tables below.

To estimate the overall comprehensiveness and sensitivity of the search string, we used a test list of documents. The initial test list used during the first scoping exercise consisted of 14 articles, all of which were captured. Since the entire test list was captured, we increased and updated the number of test articles to encounter more relevant articles and provide more comprehensive coverage across different environmental media. Therefore, we updated our list to be 24, while ensuring that all 24 documents covered all our PECO (PO) elements.

For the purpose of searching on ScienceDirect, we have conducted a search in the "Title, abstract or author-specified keywords" field. Also, ScienceDirect cannot operate like the WOSCC function, so we conducted multiple searches (search 1, search 2, etc.). (See the table below).

On the third week of July 2025, the final search string returned 190 search hits on WOSCC, with 23 out of the 24 articles of the test list indexed in the database retrieved. (See the table below)

Regarding the ScienceDirect database search, with search string number 1 (search number), 21 of the 24 articles in the test list were retrieved. However, all three missing articles were captured with the final search string using WOSCC.

| **Web of Science Core Collection (WOSCC)** | | | | | |
| --- | --- | --- | --- | --- | --- |
| **PECO*** | **Search Number** | **Search string** | **Hits** | **% of test list retrieved** | **Comments** |
| All PECO elements | 1 | TS= (("6-PPD-Q" OR "6PPD-quinone”) AND ("soil*" OR "sediment*” OR freshwater* OR "fresh water"OR runoff OR aquat* OR "particulate matter" OR "air" OR "dust")) | 140 | 75% | We have started looking for the updated test list, which includes 24. Adding more terms to the list of population and outcome combinations to see what we will get |
| All PECO elements | 2 | TS= (("6-PPD-Q" OR "6PPD-quinone”) AND ("soil*" OR "sediment*” OR freshwater* OR "fresh water"OR runoff OR aquat* OR lake*OR snow* OR ponds* OR river* "particulate matter" OR "air" OR "dust"OR "aerosol*")) | 140 | 75% | Adding more terms such as: lake*OR snow* OR ponds* OR river*OR aerosol*  Did not change anything |
| All PECO elements | 3 | TS= (("6PPD-Q" OR "6PPD-quinone”) AND ("soil*" OR "sediment*” OR freshwater* OR "fresh water"OR runoff OR aquat* OR lake*OR snow* OR ponds* OR river* "particulate matter" OR "air" OR "dust"OR "aerosol*")) | 171 | 79% | Delete the dash on the first term(6-PPD-Q), was significant.  So, 6-PPD-Q becomes 6PPD-Q |
| All PECO elements | 4 | TS= (("6PPD-Q" OR "6PPD-quinone" OR "6PPDQ”) AND ("soil*" OR "sediment*” OR freshwater* OR "fresh water" OR lake*OR snow* OR ponds* OR river* OR reservoir* OR stream* OR groundwater OR “surface water” OR runoff OR aquat* OR "particulate matter" OR "air" OR "dust" OR "aerosol*" OR "atmospher*")) | 184 | 87% | Added more technical teams such as: 6PPDQ. Also, added reservoir* OR stream* OR groundwater OR “surface water”, "atmospher*" |
| All PECO elements | 5 | TS= (("6PPD-Q" OR "6PPD-quinone" OR "6PPDQ"OR "2-anilino-5-(4-methylpentan-2-ylamino)cyclohexa-2,5-diene-1,4-dione") AND ("soil*" OR "sediment*” OR freshwater* OR "fresh water" OR lake*OR snow* OR ponds* OR river* OR reservoir* OR stream* OR groundwater OR “surface water” OR runoff OR aquat* OR "particulate matter" OR "air" OR "dust" OR "aerosol*" OR "atmospher*")) | 184 | 87% | Added the IUPAC name, did not change anything. |
| All PECO elements | 6 | TS= (("6PPD-Q" OR "6PPD-quinone" OR "6PPDQ”) AND ("soil*" OR "sediment*” OR freshwater* OR "fresh water" OR lake*OR snow* OR ponds* OR river* OR reservoir* OR stream* OR groundwater OR “surface water” OR runoff OR aquat* OR "saltwater" OR"seawater"OR"marin" OR"ocean" OR"sea"OR "particulate matter" OR "air" OR "dust" OR "aerosol*" OR "atmospher*")) | 185 | - | We added "saltwater" OR"seawater" OR"marin" OR"ocean" OR"sea", to check if the search string will cover more articles. However, it appears that it captured only one more article. So, we decided to exclude saltwater. See our eligibility criteria. |
| All PECO elements | 7 | TS= (("6PPD-Q" OR "6PPD-quinone" OR "6PPDQ”) AND ("tire wear particles" OR "tire road wear particles"OR"soil*" OR "sediment*” OR freshwater* OR "fresh water" OR lake* OR ponds* OR river* OR reservoir* OR stream* OR groundwater OR “surface water” OR runoff OR aquat* OR "particulate matter" OR "air" OR "dust" OR "aerosol*" OR "atmospher*")) | 190 | 96% | We added “tire wear tire wear particles" OR "tire road wear particles”, and that led to an increase in the results. We retrieved the missing articles that were not found in conducting search string number 5.  **This will be our final search string. However, we were unable to find one article. After a thorough search, it appears that article number 22 by Lane in our test list was not included in the WOSCC, which is why our search string was unable to locate it. Nevertheless, the article was included in the Web of Science (WOS) under the MEDLINE index.** |
|  |  |  |  |  |  |
| **ScienceDirect** | | | | | |
| **PECO*** | **Search number** | **Search string** | **Hits** | **% of test list retrieved** | **Comments** |
| O | 1 | ("6PPD-Q" OR "6PPD-quinone" OR "6PPDQ") | 157 | 88%  (3 articles are missing) | ScienceDirect uses only a maximum of 8 Boolean values for each individually running search.  **Important note: the one article was missed by WOSCC, it was captured here** |
| P+O | 2 | ("6PPD-Q" OR "6PPD-quinone" OR "6PPDQ”) AND ("tire wear particles" OR "tire road wear particles"OR"soil" OR "sediment”) | 51 | 50% | N/A |
| P+O | 3 | ("6PPD-Q" OR "6PPD-quinone" OR "6PPDQ”) AND (freshwater OR "fresh water" OR lake OR ponds OR river OR reservoir) | 25 | 33% | N/A |
| P+O | 4 | ("6PPD-Q" OR "6PPD-quinone" OR "6PPDQ")AND(freshwater OR lake OR river OR groundwater OR surface water OR runoff) | 48 | 63% | N/A |
| P+O | 5 | ("6PPD-Q" OR "6PPD-quinone" OR "6PPDQ") AND ("soil" OR "sediment" OR "air" OR "dust" OR "atmosphere" OR "water") | 66 | 83%  (4 articles are missing) | N/A |
| P+O | 6 | ("6PPD-Q" OR "6PPD-quinone" OR "6PPDQ") AND ("soil" OR "sediment" OR "air" OR "freshwater" OR "dust" OR "water") | 70 | 83%  (4 articles are missing) | N/A |
| P+O | 7 | ("6PPD-Q" OR 6PPD-quinone OR "6PPDQ") AND ("TWPs" OR "sediment" OR "air" OR "freshwater" OR "dust" OR "water") | 74 | 88  (3 articles are missing) | We deleted the quotation marks on the 6PPD-quinone and replaced the word soil with TWPs |

*: We only defined Population and Outcomes (see 4.1 Question components at the original systematic map protocol file)
