# Supplementary material for: What research exists on the presence of 6PPD-Q in different environmental media? A systematic map protocol
Source: Environ Evid. 2026 Feb 4;15:2. doi: 10.1186/s13750-026-00380-1 (PMC12922382; doi:10.1186/s13750-026-00380-1)
Supplement: Supplementary file 4 — Supplementary Material 4. [file 13750_2026_380_MOESM4_ESM.docx]

**Additional file 4: Final Search string.**

This is our final search string. The Following Search strings will be used to search in Bibliographic databases and grey literature.

- **Web of Science**

TS= (("6PPD-Q" OR "6PPD-quinone" OR "6PPDQ”) AND ("tire wear particles" OR "tire road wear particles"OR"soil*" OR "sediment*” OR freshwater* OR "fresh water" OR lake* OR ponds* OR river* OR reservoir* OR stream* OR groundwater OR “surface water” OR runoff OR aquat* OR "particulate matter" OR "air" OR "dust" OR "aerosol*" OR "atmospher*"))

- **ScienceDirect**

Search number 1

("6PPD-Q" OR "6PPD-quinone" OR "6PPDQ")

Search number 7

("6PPD-Q" OR 6PPD-quinone OR "6PPDQ") AND ("TWPs" OR "sediment" OR "air" OR "freshwater" OR "dust" OR "water")

- **BASE**

("6PPD-Q" OR "6PPD-quinone" OR "6PPDQ”) AND ("tire wear particles" OR "tire road wear particles"OR"soil*" OR "sediment*” OR freshwater* OR "fresh water" OR lake* OR ponds* OR river* OR reservoir* OR stream* OR groundwater OR “surface water” OR runoff OR aquat* OR "particulate matter" OR "air" OR "dust" OR "aerosol*" OR "atmospher*")

Note: We have selected all Text boxes in Advanced Search except the Thesis box. The search was conducted across all fields.

- **GreenFILE**

("6PPD-Q" OR "6PPD-quinone" OR "6PPDQ”) AND ("tire wear particles" OR "tire road wear particles"OR"soil*" OR "sediment*” OR freshwater* OR "fresh water" OR lake* OR ponds* OR river* OR reservoir* OR stream* OR groundwater OR “surface water” OR runoff OR aquat* OR "particulate matter" OR "air" OR "dust" OR "aerosol*" OR "atmospher*")

- **National Service Center for Environmental Publications**

("6PPD-Q" OR "6PPD-quinone" OR "6PPDQ") AND ("tire wear particles" OR "soil*" OR "sediment*" OR "air" OR "dust" OR "aerosol*" OR "atmospher*"OR “water*"OR "freshwater" OR "aqu*")

Note: We have used Boolean operators in Advanced Search and have chosen the "Exact Match" option. Everything else remains normal.

- **ProQuest Dissertations & Theses**

("6PPD-Q" OR "6PPD-quinone" OR "6PPDQ”) AND ("tire wear particles" OR "tire road wear particles"OR"soil*" OR "sediment*” OR freshwater* OR "fresh water" OR lake* OR ponds* OR river* OR reservoir* OR stream* OR groundwater OR “surface water” OR runoff OR aquat* OR "particulate matter" OR "air" OR "dust" OR "aerosol*" OR "atmospher*")

Note: We have used Boolean operators (AND) in Advanced Search. Additionally, we selected the “All abstract & summary text” option. We have applied a filter to our search for Dissertations and Theses, limiting it to English and Arabic.
